# Evaluating the Theoretical Background of STOFFENMANAGER^®^ and the Advanced REACH Tool

**DOI:** 10.1093/annweh/wxab057

**Published:** 2021-08-08

**Authors:** Antti Joonas Koivisto, Michael Jayjock, Kaarle J Hämeri, Markku Kulmala, Patrick Van Sprang, Mingzhou Yu, Brandon E Boor, Tareq Hussein, Ismo K Koponen, Jakob Löndahl, Lidia Morawska, John C Little, Susan Arnold

**Affiliations:** ARCHE Consulting, Liefkensstraat 35D, B-9032 Wondelgem, Belgium; Institute for Atmospheric and Earth System Research (INAR), University of Helsinki, PL 64, FI-00014 UHEL, Helsinki, Finland; Air Pollution Management, Willemoesgade 16, st tv, Copenhagen DK-2100, Denmark; Jayjock Associates, LLC, Langhorne, PA, USA; Institute for Atmospheric and Earth System Research (INAR), University of Helsinki, PL 64, FI-00014 UHEL, Helsinki, Finland; Institute for Atmospheric and Earth System Research (INAR), University of Helsinki, PL 64, FI-00014 UHEL, Helsinki, Finland; ARCHE Consulting, Liefkensstraat 35D, B-9032 Wondelgem, Belgium; Laboratory of Aerosol Science and Technology, China Jiliang University, Hangzhou, China; Lyles School of Civil Engineering, Purdue University, 550 Stadium Mall Drive, West Lafayette, IN 47907, USA; Ray W. Herrick Laboratories, Center for High Performance Buildings, Purdue University, 177 South Russell Street, West Lafayette, IN 47907, USA; Institute for Atmospheric and Earth System Research (INAR), University of Helsinki, PL 64, FI-00014 UHEL, Helsinki, Finland; Department of Physics, The University of Jordan, Amman 11942, Jordan; FORCE Technology, Copenhagen, Denmark; Division of Ergonomics and Aerosol Technology, Lund University, PO Box 118, SE-221 00 Lund, Sweden; International Laboratory for Air Quality and Health, Queensland University of Technology, Brisbane, QLD 4001, Australia; Global Centre for Clean Air Research (GCARE), Department of Civil and Environmental Engineering, Faculty of Engineering and Physical Sciences, University of Surrey, Guildford GU2 7XH, United Kingdom; Department of Civil and Environmental Engineering, Virginia Tech, Blacksburg, VA 24060, USA; University of Minnesota Twin Cities, Environmental Health Sciences, School of Public Health, 420 Delaware St SE, Minneapolis, MN, USA

**Keywords:** Advanced REACH Tool (ART), model evaluation, occupational exposure models, performance, REACH, regulatory acceptance, STOFFENMANAGER^®^, validation

## Abstract

STOFFENMANAGER^®^ and the Advanced REACH Tool (ART) are recommended tools by the European Chemical Agency for regulatory chemical safety assessment. The models are widely used and accepted within the scientific community. STOFFENMANAGER^®^ alone has more than 37 000 users globally and more than 310 000 risk assessment have been carried out by 2020. Regardless of their widespread use, this is the first study evaluating the theoretical backgrounds of each model. STOFFENMANAGER^®^ and ART are based on a modified multiplicative model where an exposure base level (mg m^−3^) is replaced with a dimensionless intrinsic emission score and the exposure modifying factors are replaced with multipliers that are mainly based on subjective categories that are selected by using exposure taxonomy. The intrinsic emission is a unit of concentration to the substance emission potential that represents the concentration generated in a standardized task without local ventilation. Further information or scientific justification for this selection is not provided. The multipliers have mainly discrete values given in natural logarithm steps (…, 0.3, 1, 3, …) that are allocated by expert judgements. The multipliers scientific reasoning or link to physical quantities is not reported. The models calculate a subjective exposure score, which is then translated to an exposure level (mg m^−3^) by using a calibration factor. The calibration factor is assigned by comparing the measured personal exposure levels with the exposure score that is calculated for the respective exposure scenarios. A mixed effect regression model was used to calculate correlation factors for four exposure group [e.g. dusts, vapors, mists (low-volatiles), and solid object/abrasion] by using ~1000 measurements for STOFFENMANAGER^®^ and 3000 measurements for ART. The measurement data for calibration are collected from different exposure groups. For example, for dusts the calibration data were pooled from exposure measurements sampled from pharmacies, bakeries, construction industry, and so on, which violates the empirical model basic principles. The calibration databases are not publicly available and thus their quality or subjective selections cannot be evaluated. STOFFENMANAGER^®^ and ART can be classified as subjective categorization tools providing qualitative values as their outputs. By definition, STOFFENMANAGER^®^ and ART cannot be classified as mechanistic models or empirical models. This modeling algorithm does not reflect the physical concept originally presented for the STOFFENMANAGER^®^ and ART. A literature review showed that the models have been validated only at the ‘operational analysis’ level that describes the model usability. This review revealed that the accuracy of STOFFENMANAGER^®^ is in the range of 100 000 and for ART 100. Calibration and validation studies have shown that typical log-transformed predicted exposure concentration and measured exposure levels often exhibit weak Pearson’s correlations (*r* is <0.6) for both STOFFENMANAGER^®^ and ART. Based on these limitations and performance departure from regulatory criteria for risk assessment models, it is recommended that STOFFENMANAGER^®^ and ART regulatory acceptance for chemical safety decision making should be explicitly qualified as to their current deficiencies.

**What’s Important About This Paper?**

STOFFENMANAGER^®^ and ART are widely used knowledge-based exposure models that produce qualitative exposure estimates. While these models have been evaluated at the ‘operational analysis’ level, there is a need for robust internal and external evaluations to better understand why the models tend to underestimate high exposures and overestimate low exposures. Until that is completed, the utilization of these models for REACH regulatory chemical safety assessment should be revisited.

## Introduction

STOFFENMANAGER^®^ and the Advanced REACH Tool (ART) are two of the most widely used tools for chemical safety assessment. This is because they are easy to use and have numerous features that makes the chemical safety assessment, management, and communication more reliable. Regardless of the models’ wide use and numerous publications related to their development and performance, their theoretical backgrounds are not previously evaluated. Here we evaluate the theoretical backgrounds of STOFFENMANAGER^®^ and ART and the modeling approaches taken by each.

Exposure models can be classified according to the model construct. Currently, varying terminology is used for STOFFENMANAGER^®^ and ART, which is discussed briefly, for clarity.

Models are defined by the National Research Council (NRC, 2007) as ‘a simplification of reality that is constructed to gain insights into select attributes of a particular physical, biological, economic, or social system.’, and exposure models by the World Health Organization (WHO, 2004) as ‘a conceptual or mathematical representation of the exposure process’. By following the International Programme on Chemical Safety (IPCS, 2005), exposure models can be divided into mechanistic and empirical models. Mechanistic and empirical models can be combined, where a mechanistic model uses empirical sub-models to assign values to some of the input variables. A third exposure model category can be classified as knowledge-based models. These are usually based on categories and use rules to deliver the decisions about exposure (Keil *et al.*, 2009).

**Figure 1.**
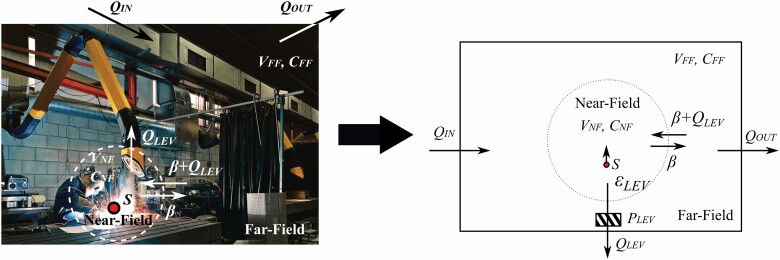
A simplified model for a welding exposure scenario. Without conservation of mass the model construction would not be possible. Reasonable model construction is not always obvious; a three-compartment model that accounts for the rising welding fume is a more appropriate model for welding emissions, as explained by Nicas *et al.* (2009) in a comment to Boelter *et al.* (2009). The two-compartment model parameters are explained in the Supplementary data Text S1, as an example of a general exposure model. The figure is modified from Koivisto *et al.* (2019b).

IPCS (2005) has defined a mechanistic model as follows ‘A mechanistic model uses process, physicochemical characteristics and mass relationships based on balance principles to predict exposures’ and ‘**Mechanistic exposure models are built on laws of physics and chemistry** and data on behaviors and factors influencing exposures — i.e. real-world exposure phenomena that are represented by equations.’ Mechanistic models are based on causal relations, which describe how physical factors are interlinked to each other. As an example of causal relations, if local exhaust ventilation is applied, it will either reduce the general ventilation exhaust volume flow rate (as air is redirected to the local exhaust) or the incoming general ventilation air volume flow is increased (so that there is air flow rate through the general exhaust is maintained, while supplemental air is directed to the local exhaust ventilation). This further influences the mass transfer from the source to the room air and reduces the exposure level. Figure 1 shows an example of how a mechanistic model can be used to simplify an environment and how the mass-balance principle is used to describe how emission from the source is translated to concentration in the room air. The relationships between parameters are described with mathematical equations that can be used to predict the source impact on concentration level at any time.

Mechanistic models can be used to calculate concentration levels even when there are no concentration measurements available, such as predicting the concentration levels for future scenarios. However, measurements are still needed for model parametrization and databases such as for sources, inter-zonal ventilation rates, and emission control efficacies are very valuable (Koivisto *et al.*, 2019a). The predicted concentration can be translated to exposure when the person exposure time and potential personal protective equipment are known.

Mechanistic models are also called as source-receptor models (Core *et al.*, 1982; IPCS, 2010; Schneider *et al.*, 2011), conceptual models (IPCS, 2005; NRC, 2007), and mass-balance models that refer to mechanistic models with some additional information (e.g. mass-balance model does not include an energy balance).

An empirical model is based on statistical associations with concentrations and other independent variables that are observed in measurement studies. These relations are then used to predict concentrations and exposure levels. Empirical models do not require or imply any causal relationships between the model variables and they do not have a physical concept (IPCS, 2005). Empirical models are especially used in descriptive data analysis (IPCS, 2005). Statistical methods can be used to identify correlating phenomena that can then studied with conceptual methods to reveal the true causal mechanisms behind the correlations. Then, when the causal mechanisms are known, process equations can be written, and mechanistic models developed to describe these relationships. According to the ICPS (2005) ‘The terms of the empirical model are specific to the data set from which they have been calculated, and there are no grounds other than expert opinion or experimental confirmation with which to assess if they can be used to calculate exposures in some other system (location/population), or even in the same system at another time.’ This limits the use of empirical models to different exposure scenarios that are well studied and parameterized.

Both mechanistic and empirical models can be classified as deterministic or probabilistic. In mechanistic model, the input variable uncertainty or range can be described with a probability density distribution. Probabilistic assessment in empirical models is based on means and standard errors of the estimated regression coefficients.

## Methods

The starting point for the study is the first introduction of STOFFENMANAGER^®^ and ART models by Marquart *et al.* (2008) and Fransman *et al.* (2013). Following the citations, we backtracked the scientific reasoning for the models. Additionally, we identified evaluation studies related to the STOFFENMANAGER^®^ and ART application in case studies, user friendliness and comparison and evaluation studies (Table 1). From these studies, terminology and developmental and theoretical considerations were extracted. Overall, these studies covered years 1996–2020 and their references. STOFFENMANAGER^®^ and ART model structures were reconstructed and the decisions and justifications regarding the parametrization and calibration were evaluated step-by-step. Reasonability of the justifications was evaluated when possible to identify their unique features, identify proper terminology, and evaluate their applicability in regulatory chemical safety decision making.

**Table 1.** Summary of the literature review and terminology used to describe STOFFENMANAGER^®^ and ART models and their predecessors.

Model	Subjective model	Mechanistic model	Other definitions
Cherrie *et al.* (1996)	Cherrie *et al.* (1996), Cherrie and Schneider (1999), Cherrie and Hughson (2005), Semple *et al.* (2001), and Van-Wendel-de-Joode *et al.* (2003)	Tielemans et al. (2008a, b)	Source-receptor: Landberg *et al.* (2017), Savic *et al.* (2017b), and Schinkel et al. (2010, 2014) Conceptual: Hesse *et al.* (2015)
Cherrie and Schneider (1999)	Cherrie and Schneider (1999), Cherrie and Hughson (2005), Lee *et al.* (2020), Semple *et al.* (2001), and Van-Wendel-de-Joode *et al.* (2003)	Cherrie *et al.* (2011), Schinkel *et al.* (2011), and Tielemans et al. (2008a, b, 2011	Conceptual: Koppisch *et al.* (2012) Mass-balance: Fransman *et al.* (2011)^b^ and Tielemans *et al.* (2008b) Source-receptor: Creely *et al.* (2005), Landberg *et al.* (2017), Lee *et al.* (2019a), Marquart *et al.* (2011), and Mc Donnell *et al.* (2011) Semi-quantitative: Marquart *et al.* (2008) and Tielemans *et al.* (2007)
**Model**	**Mechanistic model**		**Other definitions**
STOFFENMANAGER^®^	Cherrie *et al.* (2020), Huang and Wu (2019), Koppisch *et al.* (2012), Ribalta *et al.* (2019), Schinkel *et al.* (2010), and Tielemans *et al.* (2008a)		Semi-quantitative: Marquart *et al.* (2008)
ART	Cherrie et al. (2011, 2020), ECHA (2016), Fransman *et al.* (2013), Goede *et al.* (2019), Hesse *et al.* (2018), Hofstetter *et al.* (2013), Koivisto et al. (2018, 2019a), LeBlanc *et al.* (2018), Lee et al. (2019a, b), Mc Donnell *et al.* (2011), McNally *et al.* (2014), Ribalta *et al.* (2019), Riedmann *et al.* (2015), Sailabaht *et al.* (2018), Savic et al. (2016, 2017b, 2018), Schinkel et al. (2011, 2013, 2014), Schneider *et al.* (2011), Spinazzè *et al.* (2017, 2019, 2020), Tielemans et al. (2008b, 2011), and van Tongeren *et al.* (2011)		Mass-balance: Goede *et al.* (2019), Sailabaht *et al.* (2018), Tielemans *et al.* (2011) Other mechanistic model definitions that are not listed here

While training is an essential component of use, to ensure the tool is used appropriately, it is outside the scope of this assessment and will not be discussed further.

## Results

Both STOFFENMANAGER® and ART are based on a model developed by Cherrie *et al.* (1996) that involves multiplier factors. The theoretical background of the multiplicative model approach is briefly explained.

### Multiplicative models

The Cherrie *et al.* (1996) model construct is similar to a multiplicative model. Multiplicative models are empirical models based on statistical evaluation of exposure determinants in similar exposure groups. In a multiplicative model, a workplace exposure estimate, WE (mg m^−3^), is calculated by multiplying a base estimate, BE (mg m^−3^), with unitless exposure modifying factors, *K*_1_, …, *K*_*n*_. The exposure modifying factor can reflect impact of e.g. volatility or emission control to the change in the exposure level. The base estimate is a reference concentration measured in a situation where exposure modifying factors are not applied (*K*_1_ = *K*_2_ = ⋯ = 1). The simplest form of the multiplicative model is (e.g. Armstrong *et al.*, 1996)


$$\begin{array}{l} WE=BE\cdot K_{1}\cdot K_{2}\cdot \cdots \cdot K_{n} \end{array}$$ (1)

Multiplicative models can be applied appropriately for similar exposure groups where the main exposure determinants are the same and their ranges, i.e. relative weights, are known. The exposure determinants that are independent can be quantified as exposure modifying factors. Dependent exposure determinants, e.g. near-field (NF) and far-field (FF) concentrations, due to air mixing, need to be the same in different scenarios because there are no causal relations in a multiplicative model. The exposure base estimate is quantified by statistical evaluation of personal exposure levels measured in the similar exposure groups. The baseline estimate is applied to other exposure scenarios by using the exposure modifying factors that can be quantified by the following:

- Measuring exposure levels before and after a change of a single exposure modifying factor (e.g. Yu *et al.*, 1990).
- Scaling if *K* is independent from other modifying factors (e.g. Armstrong *et al.*, 1996).
- Theoretical predictions, such as Raoult’s law (Armstrong *et al.*, 1996; Lewis *et al.*, 1997).

The model reliability is strongly dependent on the level of information available for the specific case (Borghi *et al.*, 2020), similarly as in every exposure model. Multiplicative models have been applied successfully in retrospective exposure assessments for similar exposure groups (Sahmel *et al.*, 2010; Borghi *et al.*, 2020).

### Cherrie *et al.* (1996) model

In the Cherrie *et al.* (1996) multiplicative model the exposure score, *C*, is a sum of NF and FF scores:


$$\begin{array}{l} C=\varepsilon_{T,NF}\cdot t_{a,NF}\cdot \eta_{ppe}+\varepsilon_{T,FF}\cdot t_{a,FF}\cdot \eta_{ppe}\cdot d_{gv} \end{array}$$ (2)

where *C* is the dimensionless exposure score, $\varepsilon_{T}$ is the total emission, *t*_a_ is the source active time, $\eta_{ppe}$ is the personal protection factor, and *d*_gv_ is the general ventilation multiplier. The total emission is a sum of active emission *ε*_a_ and passive emission *ε*_p_:


$$\begin{array}{l} \varepsilon_{T}=\varepsilon_{a}+\varepsilon_{p}=\varepsilon_{i}\cdot h\cdot \eta_{lev}+\varepsilon_{p} \end{array}$$ (3)

where *ε*_i_ is the intrinsic emission of the material, *h* is handling or processing of the material, and *η*_lev_ is any engineering control. The physical meaning of the intrinsic emission term is not explained by Cherrie *et al.* (1996).

The multipliers have discrete values given in natural logarithm steps, as e.g. 0.1, 0.3, 1, 3, and 10. The multiplier value is subjectively selected by the user, by using an exposure taxonomy. Taxonomies provide a structure to the concepts and language used to organize knowledge (Malafsky and Newman, n.d.). Cherrie *et al.* (1996) used subjective descriptors to organize the exposure determinants. For example, a ‘very fine powder’ corresponds to *ε*_i_ = 10. This has no physical meaning, such as e.g. the very fine powder would have a dustiness index between 100 and 1000 mg kg^−1^ (Levin *et al.*, 2015).

The exposure score *C* is translated to a mass concentration (mg m^−3^) by multiplying the dimensionless exposure score with an Occupational Exposure Limit (OEL) or a similar reference value.

It can be concluded that the Cherrie *et al.* (1996) model has the following differences as compared to the multiplicative model:

1. The exposure base estimate (mg m^−3^) is replaced with an intrinsic emission multiplier and an exposure score is calculated instead of the workplace exposure estimate (WE).
2. The exposure score is translated to an exposure concentration through multiplication with an OEL. In translation, an exposure score of 1 corresponds to the OEL value.
3. The multipliers (exposure modifying factors) are not measured values but rather subjectively assigned by using the exposure taxonomy.
4. The multipliers mainly rely on discrete values assigned in natural logarithm steps.

This modeling approach is referred to a judgment-based method (Semple *et al.*, 2001). It is not a multiplicative model because the OEL has no causal relation to the occupational exposure level and the modifying factors are categories that are subjectively assigned. By definition of IPCS (2005), the Cherrie *et al.* (1996) model cannot be classified as an empirical model because there are no measurements that are statistically analyzed as e.g. in multiplicative models.

### Cherrie and Schneider (1999) model

Cherrie and Schneider (1999) is based on the Cherrie *et al.* (1996) model. In this model, the passive emission multiplier *ε*_p_ was separated from the source active time *t*_a_ as


$$\begin{array}{l} C=(\varepsilon_{i,NF}\cdot h\cdot \eta_{lev}\cdot t_{a,NF}+\varepsilon_{p})\cdot \eta_{ppe}\;\\ +(\varepsilon_{i,FF}\cdot h\cdot \eta_{lev}\cdot t_{a,FF}+\varepsilon_{p})\cdot \eta_{ppe}\cdot d_{gv} \end{array}$$ (4)

Otherwise the model parametrization and taxonomy is the same as in Cherrie *et al.* (1996).

### STOFFENMANAGER^®^ algorithm

The development of STOFFENMANAGER^®^ is presented by Tielemans *et al.* (2008a) and Marquart *et al.* (2008). The model algorithm is modified from the Cherrie and Schneider (1999) model by the following:

1. Changing the OEL to a concentration for standardized task and operational conditions that are assigned with a calibration procedure.
2. Adding a general ventilation multiplier for the NF, $\eta_{gv_{nf}}$.
3. Changing the passive emission multiplier, *ε*_p_, to a product of the intrinsic emission multiplier and a relative multiplier, *a*, for potential diffusive sources as *E a*.
4. Adding the frequency of handling *f*_h_.

By following the Marquart *et al.* (2008) nomenclature, STOFFENMANAGER^®^ calculates the subjective exposure score *B* as


$$\begin{array}{ll} B=[(E\cdot H\cdot \eta_{lc_{nf}}\cdot \eta_{gv_{nf}})+(E\cdot H\cdot \eta_{lc_{f\;f}}\cdot \eta_{gv_{f\;f}}) & \\ +(E\cdot a)]\cdot \eta_{imm}\cdot t_{h}\cdot f_{h} \end{array}$$ (5)

where the dimensionless multipliers are the intrinsic emission *E* that relates the vapor pressure of liquids and the dustiness of powders, activity emission potential *H*, localized control $\eta_{lc}$, the general ventilation multiplier $\eta_{gv}$, personal protection $\eta_{imm}$, source active time *t*_h_, and handling frequency *f*_h_. Marquart *et al.* (2008) shows the definitions and numerical ranges for each parameter. The intrinsic emission *E* was set as the same for NF, FF, and background sources to simplify the algorithms. Intrinsic emission is defined as a unit of concentration to the substance emission potential that represents the concentration generated in a standardized task without local ventilation (Cherrie and Schneider, 1999; Tielemans *et al.*, 2008b; Fransman *et al.*, 2013). Further description of the conditions in the standardized task was not provided, such as e.g. what should be the worker activity in the standardized task. STOFFENMANAGER^®^ software assumes that worker is always in the NF and that the same handling is conducted in the FF as in the NF.

According to Schinkel *et al.* (2010), the STOFFENMANAGER^®^ mechanistic concept is given by Tielemans *et al.* (2008b). It consists of six compartments (NF, FF, personal enclosure, source enclosure, a local control influence region, and surfaces; see definitions from Tielemans *et al.* 2008b), a source, and one barrier between the NF and receptor. The mass-balance concept can be described by using six coupled differential equations. However, the physical concept is unrelated to the STOFFENMANAGER^®^ exposure algorithm presented in equation (5). It is not explained how the equation (5) is derived from the concept by Tielemans *et al.* (2008b). Similarly, as with multiplicative models, STOFFENMANAGER^®^ does not have causal relations between the exposure modifying factors and it lacks a physics-based mass-balance framework.

#### Multipliers in STOFFENMANAGER^®^

The multipliers (exposure modifying factors in a multiplicative model) are categorial; the values are allocated largely by expert judgment (Marquart *et al.*, 2008). Similarly to Cherrie *et al.* (1996), the multiplier values are given in natural logarithmic steps (…, 0.3, 1, 3, …). The multipliers are quantitative (measured) and qualitative (subjectively assigned value) depending on the nature of the factor:

- Personal protection, process time, and process frequency can be considered as quantitative multipliers.
- Intrinsic emission for volatile organic compounds is based on the Raoult’s law; however, the evaporation surface area is a subjective description and is a qualitative multiplier.
- Intrinsic emission for powders is a qualitative multiplier.
- Activity emission potential is a qualitative multiplier.
- General ventilation multipliers, emission controls, relative multiplier for background emission, and separation (subgroup in a personal protection) are qualitative multipliers.

Qualitative values are assigned by expert judgements with limited and sometimes insufficient justification (Marquart *et al.*, 2008). For example, derivation of the general ventilation multipliers is not explained and it is not possible to reproduce the values from Cherrie (1999). A discussion of the uncertainty in deriving appropriate values for the general ventilation multiplier is presented in the Supplementary data Text S2.

The user can select the multiplier by following the exposure taxonomy (Marquart *et al.*, 2008). Similarly to Cherrie *et al.* (1996), the subjective multipliers are not physical quantities. The user cannot select a value for a subjective multiplier based on measured quantities (e.g. 10 mg min^−1^); selection is made by using the exposure taxonomy. The multiplier’s appropriate values cannot be verified with measurements.

Qualitative descriptors, such as ‘very small amounts’ or ‘low speed’, depend strongly on the process and the exposure scenario. For example, 10 kg can be a large quantity in pharmaceutical powder handling but a small quantity in cement mixing. Because there are no physical quantities, e.g. ‘very small amounts’ is <100 g, which increases the risk of misinterpretation when the same exposure taxonomy is used for different exposure groups (DEGs). Schinkel *et al.* (2010) and Koppish *et al.* (2012) found it challenging to assess e.g. intrinsic emission and handling score by using free-text data fields.

#### STOFFENMANAGER^®^ calibration: A quantification of the exposure score

STOFFENMANAGER^®^ quantification is based on the approach by Cherrie and Schneider (1999), but the OEL reference value was substituted for concentration in a standardized task and operational conditions (Tielemans *et al.*, 2008b; see also the definition in Fransman *et al.*, 2013). The standardized task concentration is assigned by a calibration procedure; a personal exposure level is measured for a well-specified condition and compared with the calculated exposure score *B* for the exposure scenario. A single datapoint for calibration consists of following steps:

1. A personal exposure measurement is collected, and the measurer records the contextual information.
2. Calibrators interpret the contextual information registered by the measurer and assign the multiplier values by following the exposure taxonomy. Missing information is filled in by an expert panel together with the calibrators. A subjective exposure score is then calculated by using equation (5).
3. The calibration data point is the ratio of the measured personal exposure level and the subjective exposure score *B*.
4. Calibration data are collected from DEGs that are separated into four exposure categories: (i) handling powders and granules (*n* = 408 measurements), (ii) handling resulting in comminuting (*n* = 112 measurements), (iii) handling low-volatile substances (*n* = 256 measurements), and (iv) handling volatile substances (*n* = 176 measurements).
5. Each exposure group is assigned a calibration factor by using a log-normal, mixed effect regression models, with random between- and within-company components of variance (Tielemans *et al.*, 2008a; Schinkel *et al.*, 2010).

The regression model intercept and slope are used to predict a geometric mean exposure as a function of the exposure score and between- and within-company components of variance are used to calculate the percentiles of the exposure distribution, which shape is assumed to be log-normal (Hesse *et al.*, 2015). The calibration procedure was revised by Hesse *et al.* (2015) who addressed some of the limitations related to the calibration data collection and data coverage.

The STOFFENMANAGER^®^ calibration was first performed first by Tielemans *et al.* (2008a) and was later updated by Schinkel *et al.* (2010) and Koppisch *et al.* (2012). The calibration databases are not publicly available, preventing evaluation of the data quality (Hesse *et al.*, 2015). The calibration is performed by using DEGs, which is inappropriate for multiplicative exposure modeling approaches because it blends exposure data from disparate industries, tasks, and agents. This means that e.g. pharmaceutical powder exposure score is translated to an exposure level (mg m^−3^) by using a calibration factor assigned by using exposure data from e.g. pharmacies, bakeries, construction industry, and woodworking industry (e.g. Tielemans *et al.*, 2008a). Using DEGs in calibrations is a questionable approach because the exposure modifying factors between DEGs:

1) may not be the same,
2) their sensitivities can be different, and
3) their ranges are likely different.

STOFFENMANAGER^®^ developers also found the variation of exposure determinants in DEGs to be challenging to address (Tielemans *et al.*, 2008a; Schinkel *et al.*, 2010; Koppisch *et al.*, 2012).

Common factors that required expert judgment were related to the emission source, such as intrinsic emission, activity emission potential, and background sources (Koppisch *et al.*, 2012). The source is point-of-departure for exposure. This makes it one of the most important exposure determinants and has a significant impact on the calibration factor uncertainty.

Subjective models cannot be quantified by using a calibration factor, regardless of the calibration database quality. Uncertainty or error analysis of subjectively assigned calibration factors is of questionable value, because these factors depend on the measurer’s and calibrators’ subjective opinions and interpretations. Subjective model inputs produce subjective outputs. However, to maintain consistent terminology, we will continue to refer to this procedure as a calibration.

### The ART algorithm

The development of ART is presented by Fransman *et al.* (2013). The model algorithm and allocation of multipliers is similar to STOFFENMANAGER^®^. ART was extended to include the following:

1. Segregation (*Seg*). Isolation of sources from the work environment without containment of the source itself.
2. Personal behavior (*P*). How orientation and distance of the worker to the source in the NF determines the potential exposure.
3. Separation (*Sep*). A personal enclosure for the worker within a work environment, e.g. air conditioned cabin.
4. Surface contamination (*Su*). Emission related to release of deposited contaminants on surrounding surfaces due to natural means or general workplace activities. This replaced the term for potential diffusive sources (*E a*) in STOFFENMANAGER^®^.

By following the nomenclature in Fransman *et al.* (2013), the algorithm to calculate the subjective exposure score *C*_t_ is (bold shows the new multipliers)


$$\begin{array}{l} C_{t}=[(E_{nf}\cdot H_{nf}\cdot LC_{nf}\cdot P_{nf}+Su_{nf})\cdot D_{nf}\;\\ +(E_{f\;f}\cdot H_{f\;f}\cdot LC_{f\;f}\cdot Seg_{ff}+Su_{ff})\cdot D_{f\;f}\cdot Sep_{ff}]\cdot RPE \end{array}$$ (6)

The dimensionless multipliers are substance emission potential *E*, activity emission potential *H*, localized control LC, personal behavior *P*, dilution *D*, emission from surface contamination *Su*, segregation *Seg*, and separation *Sep*. (Fransman *et al.*, 2013) shows the definitions and numerical ranges for each parameter.

ART’s physical concept is presented by Tielemans *et al.* (2008b) and Fransman *et al.* (2013). The physical concept does not have any relationship to the ART exposure algorithm presented in equation (6). ART does not have causal relations between the parameters and it is not based on an underlying physical concept.

#### Multipliers in ART

The multipliers are mainly based on categories whose values are allocated by expert judgements. An overview of the multipliers is given by Fransman *et al.* (2011) and more detailed descriptions with justifications can be found from Fransman *et al.* (2013). The values are given mainly in natural logarithm steps (…, 0.3, 1, 3, …) similarly as in Cherrie *et al.* (1996). As a short summary, ART multipliers by nature are quantitative values, quantitative categorized values (physical ranges), and qualitative values assigned by expert judgment (Fransman et al., 2011, 2013):

- *Substance emission potential multipliers for liquids* are quantitative multipliers (exact values).
- *Activity emission potential multipliers for liquid scenarios* (Marquart *et al.*, 2011) are mainly quantitative multipliers (categories), such as in spraying ‘Moderate application rate’ multiplier 1 corresponds to an application rate of (0.3–3 l min^-1^), but rely also on qualitative multipliers, such as ‘Large scale space spraying’ is 10 or ‘Careful handling’ is 0.3.
- *Substance emission potential multipliers for powders and granules* can be assigned quantitatively (categories) or qualitatively (van Tongeren *et al.*, 2011). It is worth mentioning that the dustiness index is measured in EN 17199:2019 at RH 50%. If a precautionary approach is followed, the measurement should be made for dry powders (e.g. RH < 10%; Levin *et al.*, 2015).
- *Activity emission potential multipliers for powders and granules* (Marquart *et al.*, 2011) are mainly qualitative multipliers, e.g. ‘Careful breaking stones’ is 0.3.
- *Localized controls* are quantitative multipliers (categories) based on the Exposure Control Efficacy Library (Fransman et al., 2008, 2013) and expert judgements.
- *General ventilation multipliers* are quantitative (categories) based on mathematical NF/FF model simulations (Cherrie, 1999; Cherrie *et al.*, 2011). See Supplementary data Text S2 about discussion of the errors in the general ventilation multipliers.
- Surface contamination and fugitive emissions are qualitative multipliers.
- Personal behavior is a qualitative multiplier.

Sailabaht *et al.* (2018) extended the ART multipliers for welding fumes, which are not evaluated here.

#### ART calibration: A quantification of the exposure score

The ART exposure score *C*_t_ is quantified by using a similar calibration procedure as in STOFFENMANAGER^®^ (Schinkel *et al.*, 2011). The ART estimate is predicted by a log-normal mixed effects model (Tielemans *et al.*, 2008a). While STOFFENMANAGER^®^ has two random effect variables, ART has three variabilities representing between-worker, within-worker, and between-company random effects (Tielemans *et al.*, 2011; McNally *et al.*, 2014). The variabilities of between worker and within worker were adopted from geometric standard deviation values reported by Kromhout *et al.* (1993). The between-company variability was taken from Symanski *et al.* (2006). McNally *et al.* (2014) shows examples how the statistical analysis is applied and Schinkel *et al.* (2013) gives an overview of the data base. The calibration database is not publicly accessible.

The calibration uses personal exposure measurements from DEGs to calculate calibration factors for four exposure groups: (i) Dusts, (ii) Vapors, (iii) Mists (low-volatiles), and (iv) Solid object/abrasion. The calibration factor is qualitative because ART uses subjectively assigned multipliers. Similarly, as with STOFFENMANAGER^®^, ART is deemed a non-conceptual subjective model that produces subjective exposure estimates.

### Error and uncertainty analysis

**Figure 2.**
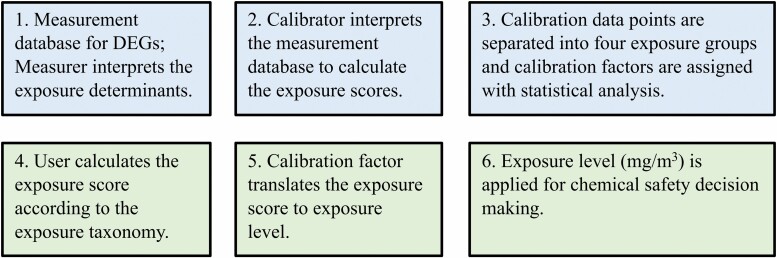
An overview of the modeling approach in STOFFENMANAGER^®^ and ART. Blue boxes illustrate the developmental part and green boxes illustrate the use part. Abbreviation: DEG, different exposure group.

The modeling approach by STOFFENMANAGER^®^ and ART is subject to errors and uncertainties that originate from both the developmental and use parts (Fig. 2). The main sources for uncertainties are related to subjective interpretation of the exposure taxonomy, categorized multiplier values, and calibration by using subjective calibration factors when

1. The measurer does not report all exposure determinants or mis-interprets the exposure taxonomy by the expert panel.
2. The calibrator, with support of the expert panel, mis-interprets the reported exposure determinants or assigns wrong values for the missing information.
3. The DEGs do not have similar exposure determinants, their relative weights are different or their ranges do not fit in the exposure taxonomy range.
4. The model user mis-interprets the exposure taxonomy as compared to the expert panel during the calibration procedure.
5. The calibration factor assigned from the DEG measurements does not represent the modeling exposure scenario.

Interpretation of the exposure taxonomy and the fixed amount of exposure determinants applied for DEGs makes the parametrization challenging even within different expert groups (Schinkel *et al.*, 2014; Spinazzè *et al.*, 2020).

The relative weights of the multipliers have not been evaluated. For example, the NF multiplier for long-term exposure to gases and vapors at 10 air exchanges per hour for a 30 m^3^ room is 3, which is the same as splash loading. It is challenging to estimate the reasonability because the multipliers rely on subjective assignment (STOFFENMANAGER^®^ and ART), quantitative exact values (ART), and quantitative categories (STOFFENMANAGER^®^ and ART). Thus, the sensitivities, i.e. impact on the exposure score, of the multipliers for the corresponding exposure scenario cannot be evaluated.

The probabilistic approach of STOFFENMANAGER^®^ and ART is based on statistical evaluation of the calibration data, i.e. the relationship between exposure scores (*B* or *C*_t_) and measured exposure concentrations (Tielemans *et al.*, 2008a; Schinkel et al., 2010, 2011; Koppisch *et al.*, 2012). Uncertainties or systematic errors of the multipliers have not been evaluated because it is not possible to perform such analyses for qualitative values. For example, in mechanistic modeling, uncertainty analysis can be performed separately for each exposure determinant that defines the result uncertainty and variation. However, this is not relevant for STOFFENMANAGER^®^ and ART because the exposure score is translated to an exposure level (mg m^−3^) by using a qualitative calibration factor. This was also addressed by Cherrie *et al.* (2020) who wrote that ‘So even if there were any minor errors made in the numerical weights for the dispersion multiplier, this will have been compensated for in the calibration with quantitative estimates of exposure,…’.

## Discussion

### Model classification

The Cherrie *et al.* (1996) and Cherrie and Schneider (1999) were referred to as subjective models until 2005 after which the models were called as mechanistic models, source-receptor models, conceptual models, mass-balance models, and semi-quantitative models (Table 1). None of the referenced studies refer to STOFFENMANAGER® or ART as subjective models, as their predecessors were originally designed.

Based on the STOFFENMANAGER^®^ and ART theoretical background, these models are not as mechanistic models by definition of IPCS (2005). Cherrie *et al.* (2020) statement ‘The mechanistic modelling that underpins both Stoffenmanager^®^ and ART are based on a theoretical analysis in which the multipliers were derived based on physical laws and data in combination with expert judgement’ is misleading. Also, the statement ‘The tools [STOFFENMANAGER^®^ and ART] were developed from conceptual source-receptor exposure models that were based on sound scientific principles (Tielemans *et al.*, 2008b)’ can be misleading, because it fails to acknowledge the disconnect between the original basis and current construct.

STOFFENMANAGER^®^ and ART calibration factors are calculated using both personal measurements and calculated subjective exposure scores from DEGs. Thus, they are not empirical models by the IPCS (2005) definition. In exposure sciences, models based on subjective categories are called judgment-based models, knowledge-based models, or expert decision tools (Cherrie *et al.*, 1996; Cherrie and Schneider, 1999; Semple *et al.*, 2001; Keil *et al.*, 2009). STOFFENMANAGER^®^ and ART models are subjective modeling approaches according to general terminology.

### Calibration of STOFFENMANAGER^®^ and ART

The subjective calibration approach and use of subjective calibration factors weakens the connection between model output and real-world exposures. Each exposure scenario should be individually calibrated because different exposure scenarios have different exposure determinants, and their magnitudes vary. It is not logical to use a single calibration factor for an entire exposure group of chemical phase or process [dusts, vapors, mists (low-volatiles), and solid object/abrasion]) because the exposure determinants can vary significantly (e.g. dusts in pharmaceutical industry and bakeries).

Use of calibration factors in exposure models is questionable. The U.S. EPA (2003) Guideline on Air Quality Models concluded that:

‘Calibration of models is not common practice and is subject to much error and misunderstanding. There have been attempts by some to compare model estimates and measurements on an event-by-event basis and then calibrate a model with results of that comparison. This approach is severely limited by uncertainties in both source and meteorological data and therefore it is difficult to precisely estimate the concentration at an exact location for a specific increment of time. Such uncertainties make calibration of models of questionable benefit. **Therefore, model calibration is unacceptable.’**

This questions the Cherrie *et al.* (2020) statement that the calibration is often considered as being the ‘gold standard’ in occupational exposure assessment. Cherrie *et al.* (2020) also suggests that the small errors in the general ventilation multipliers (Koivisto *et al.*, 2018) are compensated for by the model calibration. Calibration is not a scientifically sound method to correct model errors and subjects the exposure assessment to additional sources of error, increasing the model complexity that results in an additional factor in error analysis.

A critical component in chemical safety assessment is transparency (IPCS, 2005; NRC, 2007; EFSA, 2009; Heinemeyer *et al.*, 2019; U.S. EPA, 2019b). To ensure transparency, calibration requires traceability to nationally or internationally recognized standards. However, STOFFENMANAGER^®^ and ART calibration databases are not publicly available (Hesse *et al.*, 2015; Lee *et al.*, 2019a; Cosanta and W. Fransman, personal communication). Without transparent data analysis and calculation routines, the modeling result applicability and goodness cannot be verified. This lack of traceability and transparency associated with the subjective assignment of factors and their ‘calibration’ weaken the reliability of exposure assessments based on STOFFENMANAGER and ART. Despite not meeting these criteria, STOFFENMANAGER^®^ and ART are recommended by European Chemical Agency (ECHA) for occupational exposure assessment tools (ECHA, 2016).

### Validation of STOFFENMANAGER^®^ and ART

Different meanings exists for the term ‘validation’ (ISES Europe, 2020). Tischer *et al.* (2017) divided the validation into three parts, which were defined as the following:

1. Internal validation is testing that the model theory and computational algorithms are correct (comparison of model calculation routines with theory).
2. External validation is to evaluate whether the theory reflects the reality when all parameters are known and underlying assumptions are fulfilled (comparison of model predictions with well controlled measurements). A good example of external validation is given by Nicas (1996) for a NF/FF model.
3. Usability of the model by performing an ‘operational analysis’ (Tischer *et al.*, 2003).

The validation procedure should follow this order because if prior validation steps fail, the latter ones are of little use. STOFFENMANAGER^®^ and ART have not been internally or externally validated. In a previous study, we tried to re-calculate the general ventilation multipliers as a part of the internal validation, but they were not possible to reproduce (Koivisto *et al.*, 2018). However, internal validation of the models is not possible as long as there are unknown factors, such as the calibration databases or there are missing explanations from expert judgements (Lee *et al.*, 2019a). Neither STOFFENMANAGER^®^ or ART is challenged to a well-specified chamber experiments where all exposure determinants and their magnitudes are known (e.g. Nicas, 1996). STOFFENMANAGER^®^ and ART are validated only at the operational analysis level (Schinkel *et al.*, 2010; Mc Donnell *et al.*, 2011; Koivisto *et al.*, 2015; Lamb *et al.*, 2015; Landberg et al., 2015, 2017, 2018; Riedmann *et al.*, 2015; Bekker *et al.*, 2016; Savic et al., 2016, 2017b, 2018; Heussen and Hollander, 2017; Spinazzè *et al.*, 2017; van Tongeren *et al.*, 2017). Internal and external validations should be performed before applying them to chemical safety assessment (IPCS, 2005; NRC, 2007; Heinemeyer *et al.*, 2019; U.S. EPA, 2019a). In Supplementary data Text S3, we show an example how external validation can be performed by using experiments by Spencer and Plisko (2007).

### STOFFENMANAGER^®^ and ART predictive accuracy in operational analysis

The precision of an exposure model can be tested by simulating an exposure scenario and by comparing the simulation results with the measured exposure levels, i.e. ratio of modeled concentration to the measured concentration. STOFFENMANAGER^®^ and ART predictive accuracy has been widely studied (Lamb *et al.*, 2015; Riedmann *et al.*, 2015; Landberg et al., 2017, 2018; Savic *et al.*, 2017a; Spinazzè et al., 2017, 2020; van Tongeren *et al.*, 2017; LeBlanc *et al.*, 2018; Lee et al., 2019a, b). They show that the typical ratio of modeled and measured concentration is in the range of 100 000 for STOFFENMANAGER^®^ and 100 for ART. This means that if we have a true 1 mg m^−3^ concentration in the workplace, the concentration predicted with STOFFENMANAGER^®^ or ART ranges from 0.001 to 100 mg m^−3^ and 0.1 to 10 mg m^−3^, respectively. The precision of single or two-compartment model is typically <10, i.e. in this example the range would be from 0.5 to 2 mg m^−3^ (Jayjock *et al.*, 2011; Koivisto *et al.*, 2019a).

STOFFENMANAGER^®^ and ART calibration and validation have shown spearman correlations for log-transformed data ranging from −0.42 (volatile liquids) ≤ *r* ≤ 0.83 (powder handling) and −0.03 (spreading of liquid products) ≤ *r* ≤ 0.96 (organic solvents), respectively (see full list of correlation studies from Supplementary data Text S4). Multiple linear regression analysis showed that ART explains 49, 30, 27, and 10% of the variance for vapors, powders, wood/stone dusts, and metal dusts, respectively (Savic *et al.*, 2017a). This showed that the multipliers do not explain the exposure very well (Savic *et al.*, 2017a). Residual analysis shows that STOFFENMANAGER^®^ and ART have a systematic tendency to overestimate low exposures and underestimate high exposures (Mc Donnell *et al.*, 2011; Landberg *et al.*, 2017; Savic *et al.*, 2017a; Lee et al., 2019a, b, 2020). The reason for this trend is not known.

STOFFENMANAGER^®^ and ART are not intended to reproduce the exposure level, but rather to produce precautionary estimates of the exposure level (ECHA, 2016, p. 14; Savic *et al.*, 2017a; Landberg *et al.*, 2018; Lee *et al.*, 2019a). The aim is that <10% of the modeled values are below exposure measurements (Savic *et al.*, 2017a). This is usually achieved by using 90th percentile of the mean, which expresses exposure variability. However, because the models often do not fulfill this condition, it is recommended to use e.g. the upper level of the 90 or 95% confidence interval of the 90th or 95th percentile (Savic *et al.*, 2017a; Landberg *et al.*, 2018; Lee *et al.*, 2019a), where the confidence interval indicates the uncertainty around the percentile estimate. Lee *et al.* (2019a) shows an example how the use of higher upper percentiles and confidence intervals increases the predicted exposure levels.

The Cherrie and Schneider (1999) model validation showed correlation of 0.31 < *r* < 0.93 when the typical ratio of modeled to measured concentration was in the range of 100. Respectively, Semple *et al.* (2001) showed correlations of 0.73 < *r* < 0.85 when the typical ratio of modeled to measured concentration was in the range of 10. Semple *et al.* (2001) concluded that ‘subjective exposure modeling can be successfully used to train groups of occupational hygienists to estimate personal exposure levels’. The Cherrie and Schneider (1999) model predictability is similar or better than STOFFENMANAGER^®^ and ART. This suggests that replacement of the calibration factor with OELs or other relevant reference values may improve the performance of STOFFENMANAGER^®^ and ART.

### Regulatory acceptance

STOFFENMANAGER^®^ and ART are in line with the current EU legislation on exposure to chemical agents and chemical safety (ECHA, 2016). STOFFENMANAGER^®^ was commissioned by the Dutch Ministry of Social Affairs and Employment and is directly applicable to eleven directives by the European Commission (https://stoffenmanager.com/what-is-stoffenmanager/). The requirements for the regulatory safety decision model are scarcely explained. The Dutch Social Economic Council (Rijksoverheid) lists following criteria:

1. Twenty comparisons per application domain.
2. Evaluation is done separately for solids, liquids, and/or gases/fumes.
3. The Spearman correlation in comparison is at least 0.6.
4. The tool estimates a reasonable worst-case which represents the upper-end side of possible exposure values.
5. Measurements do not exceed the model estimates for more than 10% of the total comparisons.

The first two points are fulfilled. The correlation between modeled and measured results is partially fulfilled for log-transformed data. The models fail to estimate reasonable worst-case exposure estimates usually by two or more orders of magnitude. Because of this high uncertainty, the exposure is often underestimated in over 10% of the predicted exposures even when using the upper level of the 90 or 95% confidence interval of the 90th or 95th percentile (Landberg et al., 2015, 2017; Riedmann *et al.*, 2015; Savic *et al.*, 2017a; van Tongeren *et al.*, 2017; Lee et al., 2019a, b, 2020). The STOFFENMANAGER^®^ evaluation report by Dutch Ministry of Social Affairs and Employment is not accessible according to personal communication with Cosanta, ECHA, The Finnish Safety and Chemicals Agency, and Rijksoverheid (the Netherlands).

One criterion that Raul and Dwyer (2003) used to assess whether expert witnesses’ scientific testimony is methodologically valid is the Daubert standard (Daubert v. Merrell Dow Pharmaceuticals, Inc., 509 U.S. 579, 1993). The standard provides five criteria that may be used to assess the validity of the methodology, which the STOFFENMANAGER^®^ and ART occupational exposure models partially fulfill (Table 2). As proposed earlier (Koivisto et al., 2019a, b), the rationale for STOFFENMANAGER^®^ and ART should be reviewed similarly as it has been done for the two-compartment model by Jayjock *et al.* (2011) and by following good practices (e.g. NRC, 2007; U.S. EPA, 2009, 2019b).

**Table 2.** Daubert criteria and the compliance of STOFFENMANAGER^®^ and ART.

Daubert criteria	Compliance
Is applicable and has been tested	The models have been validated and tested only at ‘operational analysis’ level
Has been subjected to peer review and is generally accepted	Calibration database is not subjected to peer review and this is the first study evaluating the theoretical background in detail. It is the scientific community’s responsibility to evaluate findings in this study and decide if the models constructs are acceptable for regulatory chemical safety decision making
The rate of error is known and acceptable, i.e. ‘Does the chosen model, with its simplifying assumptions, adequately simulate conditions to give reasonable estimates and useful insights?’ (Jayjock *et al.*, 2011)	The rate of error has been shown very high. The models have shown high uncertainty why their applicability in a chemical safety decision making should be revised
The existence and maintenance of standards and controls concerning the operation	The models fulfills this condition
Is generally accepted in the relevant scientific community	This should be revised by including the findings from this study and the calibration data bases

### Impact on the REACH regulatory chemical safety assessment

ECHA R.14 (2016) recommends estimating exposure by starting with tier 1 modeling and, on the basis of the results, identify a limited number of (contributing) scenarios for which either higher tier modeling or a measurement is needed. The tiered exposure assessment approach is an iterative process that ends when the information level is acceptable for the chemical safety decision making (Tielemans *et al.*, 2007). A condition for the tiered approach is that lower tier level always results in a higher exposure estimate (Keil, 2000; Tielemans *et al.*, 2007; Heinemeyer *et al.*, 2019). Usually, the lowest tier model is a parametrization corresponding to a theoretical worst-case exposure estimate. Supplementary data Text S1 shows an example of a tiered approach parametrization for a mass-balance model that is always favoring higher exposure estimates at lower tier levels.

STOFFENMANAGER^®^ and ART are classified as tier 1.5 and 2 models, respectively. However, the models do not follow the precautionary principle; ART can underestimate the exposure more than STOFFENMANAGER^®^ depending on the exposure scenario. As an example, Spinazzè *et al.* (2017) showed that STOFFENMANAGER^®^ (90th percentile) produced lower exposure estimates than ART (90th percentile; 95th confidence interval) for the same exposure scenario. It was also shown that the predicted exposure estimates were below the measured exposure level in 9 out of 32 cases for STOFFENMANAGER^®^ and 15 out of 35 cases for ART. Landberg *et al.* (2017) and Lee *et al.* (2019a) made similar findings for STOFFENMANAGER^®^ and ART. This mean that in some cases STOFFENMANAGER^®^ produced lower exposure estimates compared to ART, i.e. STOFFENMANAGER^®^ was less precautionary than ART. Similar observations were seen in the external validation example (Supplementary data Text S3).

Combining the model’s high uncertainties with subjective interpretation of exposure taxonomy and a tiered approach can produce nearly any outcome. These factors thus make the model outcomes vulnerable to misalignment relative to reality and makes vulnerable to misuse, if the subjective selections and interpretations are manipulated to produce a desired outcome. STOFFENMANAGER^®^ and ART user-friendly designs mean that modeling can be performed without any measurements; parametrization can be assigned by using an exposure taxonomy and information provided in the material safety data sheet. This makes STOFFENMANAGER^®^ and ART currently the most powerful tools in the REACH chemical safety assessment.

These issues do not apply to mechanistic models because validity of the model physical concept and physical parametrization can be verified. Mechanistic modeling requires a basic understanding of the exposure determinants, such as the source emissions. The need to have a minimum level of understanding the exposure scenario is one of the most important precautionary actions in chemical safety assessment based on modeling. This requirement is missing from STOFFENMANAGER^®^ and ART where the modeler does not need to understand physical parameters and the meanings of magnitudes (e.g. ‘very fine powder’).

### Mechanistic models as an alternative option

Numerous mechanistic models exist for predictive exposure assessment (Supplementary data Text S1). The future development in exposure sciences depends on understanding proper model parametrization, such as process emissions, emission controls, mixing of pollutants, and human behavior (Koivisto *et al.*, 2019a). Default exposure determinant databases are needed for different industrial processes and sectors as have been built for consumer exposure (Meesters *et al.*, 2018; U.S. EPA, 2018).

## Conclusions

STOFFENMANAGER^®^ and ART have been validated at the ‘operational analysis’ level. However, their theoretical backgrounds have not been validated and the models’ structures and parametrizations are not well understood. Here we evaluated the STOFFENMANAGER^®^ and ART model development step-by-step starting from the very beginning, with a multiplicative model that is a foundation of the models, and concluding with their applicability in regulatory chemical safety assessment.

STOFFENMANAGER^®^ and ART are not mechanistic models as defined by the International Programme on Chemical Safety, because (i) they are not dependent on an underlying physical concept with causal relationships between the exposure determinants; (ii) their parametrizations rely on categorized values that are partially assigned by using a subjective exposure taxonomy; and (iii) the models are calibrated by using a qualitative calibration factor that is subjectively assigned from DEG measurements. Calibration by using DEGs is not in line with ECHA requirements and EN 689 exposure assessment, where only similar exposure groups can be grouped for statistical evaluation. The use of DEGs violates the basic principles of empirical modeling.

STOFFENMANAGER^®^ and ART are subjective categorization tools regardless of whether the output is given in physical units (mg m^−3^). Subjective models produce subjective outputs that cannot be quantified with the calibration procedure.

Recent studies have shown that the predictability of exposure, i.e. ratio of modeled to measured concentrations, is in the range 100 000-fold for STOFFENMANAGER^®^ and in a range of 100-fold for ART. A tiered approach combined with STOFFENMANAGER^®^ and ART can be used to generate modeling outputs according to the user needs so that the parametrization meets the real conditions. STOFFENMANAGER^®^ and ART do not fulfill the Dutch Social Economic Council requirements for regulatory models or the Daubert standard for methodological validation. Although ECHA recommends STM and ART, it is necessary to be aware of their uncertainties in chemical safety assessment. This contradicts the quantitative exposure estimates requirement in ECHA R.14 (2016). Regulatory exposure models should be transparent and expert judgements, calibration databases, and the regulatory acceptance commissioned by the Dutch Ministry of Social Affairs and Employment should be publicly available.

## Supplementary Material

wxab057_suppl_Supplementary-Material

## Data Availability

No data were used in this study.
