# Supplementary material for: Evaluating the Theoretical Background of STOFFENMANAGER® and the Advanced REACH Tool
Source: Ann Work Expo Health. 2021 Aug 8;66(4):520–36. doi: 10.1093/annweh/wxab057 (PMC9030124; doi:10.1093/annweh/wxab057)
Supplement: wxab057_suppl_Supplementary-Material [file wxab057_suppl_supplementary-material.pdf]

**SUPPLEMENTARY MATERIAL**

# Evaluating the Theoretical Background of STOFFENMANAGER® and the Advanced REACH Tool

Antti Joonas Koivisto[1,2,3,*], Michael Jayjock[4], Kaarle J. Hämeri[2], Markku Kulmala[2], Patrick Van Sprang[1], Yu Mingzhou[5], Brandon E. Boor[6,7], Tareq Hussein[2,8], Ismo K Koponen[9], Jakob Löndahl[10], Lidia Morawska[11,12], John C. Little[13], Susan Arnold[14]

[1]ARCHE Consulting, Liefkensstraat 35D B-9032 Wondelgem, Belgium

[2]Institute for Atmospheric and Earth System Research (INAR), University of Helsinki, PL 64, FI-00014 UHEL, Helsinki, Finland

[3]Air Pollution Management, Willemoesgade 16, st tv, Copenhagen DK-2100, Denmark

[4]Jayjock Associates, LLC, Langhorne, Pennsylvania, United States

[5]Laboratory of Aerosol Science and Technology, China Jiliang University, Hangzhou, China

[6]Lyles School of Civil Engineering, Purdue University, 550 Stadium Mall Drive, West Lafayette, Indiana 47907, United States

[7]Ray W. Herrick Laboratories, Center for High Performance Buildings, Purdue University, 177 South Russell Street, West Lafayette, Indiana 47907, United States

[8]Department of Physics, The University of Jordan, Amman 11942, Jordan

[9]FORCE Technology, Copenhagen, Denmark

[10]Department of Design Sciences, Lund University, P.O. Box 118, SE-221 00 Lund, Sweden

[11]International Laboratory for Air Quality and Health, Queensland University of Technology, Brisbane, QLD 4001, Australia

[12]Ingham Institute of Applied Medical Research, Liverpool, NSW 2170, Australia

[13]Department of Civil and Environmental Engineering, Virginia Tech, Blacksburg, Virginia 24060, United States;

[14]University of Minnesota Twin Cities, Environmental Health Sciences, School of Public Health, 420 Delaware St SE, Minneapolis, MN, United States

*Author to whom correspondence should be addressed. Tel: +32 92 16 70 37; e-mail: joonas.koivisto@arche-consulting.be

**Table of contents**


**Text S1.** Mechanistic exposure models 2

**Text S2.** The general ventilation multipliers 5

*2.1 The near-field multiplier* 5

*2.2 The far-field multiplier* 6

*2.3 Differences between the general ventilation multipliers* 6

*2.4 Summary of the general ventilation multipliers* 8

**Text S3.** External validation of NF/FF model, STOFFENMANAGER® and ART 8

**Text S4.** Summary of STOFFENMANAGER® and ART studies reporting correlation coefficients 11

**References** 13


## Text S1. Mechanistic exposure models

There are several mechanistic exposure assessment models whose complexity varies from multi-compartment dispersion models, such as e.g. PANDORA (Abadie and Blondeau, 2011), MOEEBIUS (Santos et al., 2017) or CONTAM (Dols and Polidoro, 2015) to single- or two- compartment models, such as e.g. IH-MOD 2.0 (https://ihmod.org/), TEAS (https://www.easinc.co/), GuideNano (https://tool.guidenano.eu/), ConsExpo (https://www.rivm.nl/en/consexpo) and Consumer Exposure Model (CEM; U.S. EPA, 2019). The mechanistic modeling approach is internationally recognized as the general approach in consumer exposure assessment by using one, two or three compartment models (Koontz and Nagda, 1991; SCCS, 2018; Steiling et al., 2018, 2014; U.S. EPA, 2019, 2018). CEM has also a model for gas-particle partition that is useful in exposure assessment of volatile organic compounds, which could be useful also in occupational exposure modeling's.

A multi-compartment model can always be simplified to cover exposure scenarios where there is limited amount of information. One example of the model parametrization is presented by Nymark et al. (2020). A General Exposure Model (GEM) can include relevant processes such as a local exhaust ventilation, ventilation air re-circulation, coagulation and deposition of the particles (Figure S1); in addition, it can incorporate risk management measures and calculation of regional deposited dose during inhalation. The GEM returns concentration levels and mass flows to surfaces, outdoors and filters in unit/min. Supplemental Table S1 shows one proposal for the GEM tiered parameterization depending on the process and use environment knowledge level. The model geometrical layouts should be adjustable making it applicable to predict personal exposure outdoors by setting near-field volume ($V_{FF}$) very large and inter-zonal volume flow ($\beta$) to high. The various parameters suggested per Stage should be considered for implementation into HRA tools.

The modeling can be started by one variable (emission), which is the most critical exposure determinant, and then the complexity can be increased when there is more information available from the emissions, emission control and expected use environment. The model boundaries, parameterization and assumptions are visible and their reasonability could be evaluated by the modeller. Such modeling approach can cover all tiers in the ECHA recommended exposure modeling approach. Also, it would be possible to classify the tier level according to the model concept and parametrization. Currently, the

ECHA models tier classes are not scientifically justified. This approach can be conducted with any mechanistic exposure model.

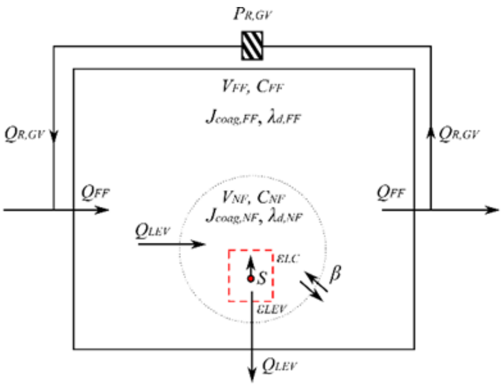


| Term | Unit | Description |
|---|---|---|
| $S$ | X min$^{-1}$ | Particle source |
| $C_{FF}$ | X m$^{-3}$ | Far field concentration |
| $C_{NF}$ | X m$^{-3}$ | Near field concentration |
| $V_{FF}$ | m$^3$ | Far field volume |
| $V_{NF}$ | m$^3$ | Near field volume |
| $\beta$ | m$^3$ min$^{-1}$ | Inter-zonal volume flow |
| $Q_{FF}$ | m$^3$ min$^{-1}$ | General ventilation volume flow |
| $\varepsilon_{LC}$ | - | Efficiency of the local control (enclosure) |
| $\varepsilon_{LEV}$ | - | Efficiency of the LEV |
| $Q_{LEV}$ | m$^3$ min$^{-1}$ | LEV volume flow |
| $\varepsilon_{R,GV}$ | - | Efficiency of the general ventilation re-circulation filter |
| $Q_{R,GV}$ | m$^3$ min$^{-1}$ | Re-circulation air volume flow |
| $J_{coag}$ | X m$^{-3}$ min$^{-1}$ | Particle coagulation rate |
| $\lambda_d$ | X m$^{-3}$ min$^{-1}$ | Particle deposition rate |

Mass balance of the model:

$$V_{FF}\frac{dC_{FF}}{dt} = \beta \cdot C_{NF} - (\beta + Q_{LEV} + Q_{FF})C_{FF} + Q_{R,GV} \cdot \varepsilon_{R,GV} \cdot C_{FF} + J_{coag,FF} \cdot C_{FF} - \lambda_{d,FF} \cdot C_{NF}$$

$$V_{NF}\frac{dC_{NF}}{dt} = (1 - \varepsilon_{LEV})(1 - \varepsilon_{LC}) \cdot S + (\beta + Q_{LEV})C_{FF} - (\beta + Q_{LEV})C_{NF} + J_{coag,NF} \cdot C_{NF} - \lambda_{d,NF} \cdot C_{NF}$$

**Figure S1.** Mass flow scheme of the GEM including enclosure, local exhaust ventilation, and re-circulation of general ventilation air.

**Table S1.** Tiered approach given by Nymark et al. (2020). Abbreviations: X is units in number, surface area (μm$^2$/m$^3$), or mass (μg/m$^3$), WC = worst case, Mo = modelled, Me = measured, DP = Default parameterization, ECEL = Exposure Control Efficacy Library, - = excluded from the model, + = included in the model.

| **Tier** | **Variables** | | | | | | | | | | **Processes** | |
|---|---|---|---|---|---|---|---|---|---|---|---|---|
| | $S$, [X s$^{-1}$] | $V_{FF}$, [m$^3$] | $V_{NF}$, [m$^3$] | $\beta$, [m$^3$s$^{-1}$] | $Q_{FF}$, [m$^3$s$^{-1}$] | $\varepsilon_{LC}$ [-] | $\varepsilon_{LEV}$, [-] | $Q_{LEV}$, [m$^3$s$^{-1}$] | $\varepsilon_{R,GV}$, [-] | $Q_{R,GV}$, [m$^3$s$^{-1}$] | $J_{coag}$, [m$^3$ s$^{-1}$] | $\lambda_d$, [m$^3$ s$^{-1}$] |
| 1 | WC | 20 | 8 | 20[a] | 0 | 0 | 0 | 0 | 0 | 0 | - | - |
| 2 | WC/Mo | 20 | 8 | 20[a] | WC | 0 | 0 | 0 | 0 | 0 | - | - |
| 3 | WC/Mo | WC/DP | WC/DP | WC/DP | WC/DP | WC/DP | WC/DP | WC/DP | 0 | 0 | - | - |
| 4 | Mo/Me | DP | DP | DP | DP | ECEL | ECEL | ECEL | DP | DP | -/+ | -/+ |
| 5 | Mo/Me | DP/Me | DP/Me | DP/Me | DP/Me | ECEL | ECEL | ECEL | DP/Me | DP/Me | + | + |

[a]Fully mixed equaling to a single box model

**Text S2. The general ventilation multipliers**

Koivisto et al. (2018) found that the general ventilation multipliers in STOFFENMANAGER® and ART were not properly calculated. This raised a concern about the models' theoretical backgrounds (Koivisto et al., 2019a). Cherrie et al. (2020) replied to the criticism but their response left many open questions. The discussion was made to continue via private communication which outcomes would be presented in a workshop "Theoretical Background of Occupational Exposure Models" organized by the ISES Europe Exposure Models Working Group. However, the dialog prior the workshop was partially successful and the workshop focus was shifted from theoretical background evaluation to other relevant issues. A majority of the scientific questions was left open (ISES Europe, 2020) and STOFFENMANAGER® and the ART development group considered the discussion closed. However, theoretical background evaluation of the regulatory exposure assessment models should end when it is accepted within the scientific community and the underlying limitations are communicated transparently to the model users and regulatory bodies. Here we tried to re-calculate the general ventilation multipliers by Marquart et al. (2008) and Cherrie et al. (2011).

The general ventilation multipliers are always present in STOFFENMANAGER® and ART modelings (Cherrie et al., 2011; Cherrie, 1999). Koivisto et al. (2018) re-calculated the general ventilation multipliers and found some differences between the numbers by Cherrie (1999) and Cherrie et al. (2011). However, Cherrie et al. (2020) did not agree with Koivisto et al. (2018) calculations but they did not specify how to calculate the values correctly. Revision of Koivisto et al. (2018) Table S2 produced the same results. However, as Koivisto et al. (2018) wrote, they were not sure how the normalization was made. They asked for the authors help, but they could not confirm the calculations. Here we have summarized main guidelines for the calculations by Cherrie (1999) and Cherrie et al. (2011).

The general ventilation multipliers are based on two compartment modelings, where Near-Field (NF) and Far-Field (FF) concentrations are calculated by using a source of 100 mg/min and a NF volume of 8 $m^3$ and then room volume ($V_{FF}$, $m^3$), air changes per hour ($ACH$, $h^{-1}$) and air mixing between NF and FF ($\beta$, $m^3$ $min^{-1}$) are varied. Cherrie (1999) calculated a reference concentration as:

- "*A large room (3000 $m^3$) with a moderate air exchange rate (three air-changes per hour)*" and this was amended in Figures 1 and 2 by defining "*intermediate airflow out of the near-field* [$\beta$ = 10 $m^3$ $min^{-1}$]". This corresponds to NF concentration of 10.6 mg $m^{-3}$.

Then, the multipliers were calculated as:

- *The near-field multiplier* was obtained by taking *the average relative near-field concentration* for the room sizes and ventilation conditions described in the table [Table IV in Cherrie (1999)], *re-normalized to the average value for large rooms with good ventilation*.
- *The far-field multiplier* was then calculated by dividing the near-field multiplier by *the average ratio of the near- to far-field concentration* obtained in the simulation.

*2.1 The near-field multiplier*

It was not specified if *the average relative near-field concentration* was normalized with the reference room NF concentration of 10.6 mg $m^{-3}$, but if normalized with 10.6 mg $m^{-3}$, the relative concentration in the NF at Cherrie (1999) does not produce the same results when using the NF or FF concentrations given by Koivisto et al. (2018) (Table S2). However, as Koivisto et al. (2018) wrote, "*it is expected that the normalization factors are constant and the same for both NF and FF concentrations in Cherrie's calculations and thus do not affect to the concentration ratios.*" Because, Koivisto et al. (2018) could not find the first normalization factor and the results in Table S2 were not consistent, they first assumed it as 10. However, later they found that the assumption was wrong and wrote "*the relative NF and FF*

*concentrations given by Cherrie (1999) are on average 9.6±0.8 and 9.1±1.4 times smaller than the respective NF and FF concentrations calculated in this study.*"

**Table S2.** Relative NF (*$RC_{NF}$*) and FF (*$RC_{FF}$*) concentrations from Cherrie (1999) Table II and calculated by using Koivisto et al. $C_{NF}$ and $C_{FF}$ values that are normalized with 10.6 mg m$^{-3}$ and ratio of NF to FF concentration ($C_{NF}/C_{FF}$). An example of six first rows of Cherrie (1999) Table II and Koivisto et al. (2018) Table 1.

| $V_{FF}$, (m$^3$) | *ACH*, (h$^{-1}$) | *β*, (m$^3$ min$^{-1}$) | Cherrie (1999), Table II | | Koivisto et al. (2018) | | $C_{NF}/C_{FF}$ |
|---|---|---|---|---|---|---|---|
| | | | $RC_{NF}$, (-) | $RC_{FF}$, (-) | $RC_{NF}$, (-) | $RC_{FF}$, (-) | |
| 30 | 0.3 | 3 | 39 | 37 | 33 | 31 | 1.1 |
| 30 | 0.3 | 10 | 37 | 37 | 32 | 31 | 1.0 |
| 30 | 0.3 | 30 | 37 | 37 | 31 | 31 | 1.0 |
| 30 | 1 | 3 | 20 | 17 | 16 | 13 | 1.2 |
| 30 | 1 | 10 | 18 | 17 | 14 | 13 | 1.1 |
| 30 | 1 | 30 | 17 | 17 | 13 | 13 | 1.0 |

The other normalization factor was *re-normalized to the average value for large rooms with good ventilation*, which is assumed to be average of simulations with $V_{FF}$ is 1000 m$^3$ and 3000 m$^3$ and ACH is 10 h$^{-1}$ and 30 h$^{-1}$. In Cherrie (1999) Table II is repeated multiple times same scenario (see e.g. last three rows in Cherrie's Table II and scenarios with $V_{FF}$ =1000 m$^3$ and ACH = 10 and 30 h$^{-1}$). Koivisto et al. (2018) did not repeat the scenarios because it overweighs some scenarios.

It can be concluded that the construction of the re-normalization factor is unclear.

Later, Cherrie et al. (2011) stated that "*Following the work of Cherrie (1999), the calculated concentration was normalized to the concentration in the NF of a 1000-m$^3$ room with 10 air changes per hour (ACH).*" However, regardless of different room conditions, the normalization concentration is the same as in Cherrie (1999) when using the NF and FF concentrations calculated by Koivisto et al. (2018).

*2.2 The far-field multiplier*

As an example, *the average ratio of the near- to far-field concentration* for small room with low ventilation is an average of 1.1 for 30 m$^3$ room with $ACH \leq 1$ h$^{-1}$ (see Table S2) and 1.2 for 100 m$^3$ room with $ACH \leq 1$ h$^{-1}$, i.e. (1.1+1.2)/2 = ~1.2. Then, the far-field multiplier is calculated by dividing the respective near-field multiplier with 1.2. The scientific justification for the normalization is not given.

*2.3 Differences between the general ventilation multipliers*

Cherrie et al. (2020) stated that "*Koivisto et al. (2018) incorrectly presents the differences between their calculations and those in the later paper (Cherrie et al., 2011), which were on average about 5% higher, a difference that we believe could reasonably be explained by small differences in the calculation methods.*" Such high difference cannot be explained by calculation methods, if correctly calculated, and they also forgot to mention that the error is not linear; depending on the room size the difference between multipliers vary from 0% to 17% for 1-h exposure estimation and from 0% to 41% for 8-h exposure. Reviewers of the Koivisto et al. (2018) manuscript also came to the same conclusion as the authors. Thus, for transparency, it would be helpful if Cherrie et al. could show their calculations, as was done in Koivisto et al. (2018).

The other statement was "*For Stoffenmanager®, the categorization of parameters and the allocation of scores for categories were partly taken from the work by Cherrie and colleagues (Cherrie et al., 1996;*

*Cherrie, 1999), but were not directly translated into Stoffenmanager® as Koivisto et al. (2018) assumes. The scores for reduction by general ventilation both for near-field and far-field sources—dependent on room size—were modified to construct a simpler model as described in Marquart et al. (2008) and Tielemans et al. (2008). Thus, Koivisto et al. (2018) are not appropriately comparing the multiplier values actually used in ART and Stoffenmanager® with their own multiplier calculations and the claim of error is unsubstantiated.*"

Koivisto et al. (2018) has taken the normalization with room size into account. This was clearly written as: "*The general ventilation multipliers were calculated as "The NF multiplier was obtained by taking the average relative NF concentration for the room sizes and ventilation conditions described in the table, re-normalized to the average value for large rooms with good ventilation. The FF multiplier was then calculated by dividing the $NF_{multiplier}$ by the average ratio of the NF to FF concentration obtained in the simulation.*". If we would not have taken this into account, the difference between multipliers would have been significantly higher.

Tielemans et al., (2008) and Marquart et al. (2008) mention that "*These scores* [general ventilation multipliers] *are related to the room volume and are taken from Cherrie (1999), who based the values on simulations.*" It is not described clearly as to which table they refer. Koivisto et al. (2018) assumed it was Table IV and did not calculate how Cherrie (1999) errors influence the Table III values.

It is unclear if Cherrie (1999) used the repeated simulation values in Cherrie's Table II for calculating the Ratio of FF to NF concentrations for ≥1000 $m^3$ & ≥ 10 ACH scenario. At least, the numbers are different when re-calculating the Ratio of FF to NF concentration using Cherrie's Table II values, as shown here in Table S3. By following digit rounding rules, the multiplier for scenario ≥1000 $m^3$ & ≤ 1 ACH should be rounded from 0.19 to 0.1 and not 0.3.

The values are slightly different when calculating the Ratio of FF to NF concentrations by using Koivisto et al. (2018) multipliers.

**Table S3.** Comparison of general ventilation model parameters and simulation results between Cherrie (1999) by using values by Koivisto et al. (2018). Bolded values differ from Cherrie (1999) Table III.

| **Simulation conditions** | ***$d_{gv}$,*** **Cherrie (1999)** | **Ratio of FF to NF concentration, Cherrie (1999) Table III** | | | **Re-calculated ratio of FF to NF concentrations by using Cherrie (1999) Table II values** | | |
|---|---|---|---|---|---|---|---|
| | | **Mean** | **min** | **max** | **Mean** | **min** | **max** |
| **≥1000 $m^3$ & ≥ 10 ACH** | 0.1 | 0.06 | 0.04 | 0.15 | **0.05** | 0.04 | 0.15 |
| **≥1000 $m^3$ & ≤ 1 ACH** | 0.3 | 0.35 | 0.05 | 0.76 | **0.19** | 0.05 | **0.77** |
| **≤ 100 $m^3$ & ≥ 10 ACH** | 0.3 | 0.42 | 0.18 | 0.83 | **0.33** | **0.19** | 0.83 |
| **≤ 1000 $m^3$ & ≤ 1 ACH** | 1 | 0.89 | 0.62 | 1.0 | **0.88** | **0.63** | 1.0 |

Finally, Table S4 shows the multipliers used in STOFFENMANAGER® (Marquart et al., 2008) that are completely different from Cherrie (1999) values. It is not explained how these multipliers have been assigned. The link between Cherrie (1999) and multipliers in Marquart et al. (2008) is unclear.

**Table S4.** Scores for reduction by general ventilation for near-field sources, dependent on room size.

| Room size | Marquart et al. (2008), Near-Field [a] | Marquart et al. (2008), Far-Field [b] | Cherrie (1999), Table III |
|---|---|---|---|
| **Volume <100 $m^3$** | 3 | 3 | 1 |
| **Volume 100–1000 $m^3$** | 1 | 0.3 | Not given |
| **Volume >1000 $m^3$** | 1 | 0.1 | 0.1 |

[a]Table 5 and [b] Table 6 in Marquart et al. (2008)

*2.4 Summary of the general ventilation multipliers*

The calculation of general ventilation multipliers and their scientific justification are far from clear. The application of general ventilation multipliers in STOFFENMANAGER® is not explained (e.g. Table S2). It is misleading say the general ventilation multipliers in STOFFENMANAGER® and ART are derived from two compartment model simulations by Cherrie (1999).

The general ventilation multiplier errors and unclear definitions are still present even though STOFFENMANAGER® and ART are, as using Cherrie et al. (2020) words, "*…extensively documented in peer-reviewed scientific papers and associate technical reports, which are available from the tool websites (https://stoffenmanager.com/what-is-stoffenmanager/; https://advancedreachtool.com/science.aspx).*" The reason for this is still the same relative to what Koivisto et al. (2018) wrote "*We believe that the errors found here would have been revealed much earlier if the tools would rely, e.g., on a standard NF/FF* [two-compartment] *model.*" It is simply not possible to validate a model without physical concept and rely on subjectively assigned multipliers.

**Text S3.** External validation of NF/FF model, STOFFENMANAGER® and ART

Here we present an example of external validation. Spencer and Plisko (2007) measured concentrations in Near-Field (NF) and Far-Field (FF) when one hundred mL of reagent-grade (100%) cyclohexane (CAS: 110-82-7) were squirted from a Nalgene laboratory wash bottle onto a 5.08 cm, Class 125 Iron Body Gate Valve during disassembly of the valve. The room air movement intensity was changed with an external fan to simulate different work environments: 1) an enclosed area or shop with little ventilation, 2) the same area or shop with open doors/windows and/or people walking in the vicinity and 3) a well-ventilated or semi-outdoor work environment. Here, we use the study to externally validate STOFFENMANAGER® and ART and compare the results with the NF/FF model external validation by Spencer and Plisko (2007).

The model parametrization was performed according to the given contextual information by Spencer and Plisko (2007). The modeling results were compared with measured NF concentrations where concentration ratios <1 underestimate the exposure and >1 overestimate the exposure. Table S5 shows that NF concentration by:

- The NF/FF model underestimated 28% in high air intensity scenario but otherwise predicted well (within 1-3%).
- STOFFENMANAGER® underestimated by 15% to 51% when using the 50$^{th}$ percent percentile.
- ART overestimated by 851 to 1460% when using the 75$^{th}$ percentile (50$^{th}$ percentile is not reported).

The external validation results shows that a NF/FF model predicts the NF concentration with expected accuracy when properly parametrized (Jayjock et al., 2011). STOFFENMANAGER® underestimates the NF concentration despite being classified as a Tier 1.5 model that is considered more precautionary than a Tier 2 model. ART overestimates significantly the NF concentration when it should give lower estimates than lower tier models, such as STOFFENMANAGER® (Tielemans et al., 2007). It can be concluded that by following ECHA recommended Tiered Exposure Assessment, the assessor can use STOFFENMANAGER® and ART to iterate an exposure assessment result that is satisfying.

Similar external evaluations should be performed especially for tasks under different operational conditions in order to identify sources and environmental conditions that violates the underlying assumptions in the NF/FF model.

**Table S5.** External validation of NF/FF model, STOFFENMANAGER® and ART by using chamber measurements by Spencer and Plisko (2007).

| Parameter | NF/FF model | STOFFENMANAGER® | ART |
|---|---|---|---|
| Simulated task description | One hundred mL of reagent-grade (100%) cyclohexane (CAS: 110-82-7) were squirted from a Nalgene laboratory wash bottle onto a 5.08 cm, Class 125 Iron Body Gate Valve during disassembly of the valve. Process temperature is 20 ºC. See details from Spencer and Plisko (2007). | | |
| Working practices (relevant for STOFFENMANAGER® and ART. | Worker is assumed to be in the NF. Emission controls or personal protective equipment is not used. There are no other workers carrying tasks and the task is not followed by a period of evaporation, drying or curing. The working room is cleaned daily. Machines/ancillary equipment are in good condition and functioning properly. | | |
| Exposure duration | 60 minutes (complete evaporation of cyclohexane) | | |
| Task repetitions | 1 | | |
| Room volume | 113 $m^3$ | 100 to 1000 $m^3$ | 100 $m^3$ (category) |
| Room ventilation rate in air changes per hour (ACH) | 4.3 ACH | General ventilation (mechanical) | 3 ACH (category) |
| NF shape | Hemisphere | Cube (fixed) | Cube (fixed) |
| NF volume | 2.1 $m^3$ (radius 1 m) | 8 $m^3$ (fixed) | 8 $m^3$ (fixed) |
| NF-FF airflow rate ($\beta$) | 10.34 $m^3$/min (low) | Not specified (see Text S2, Supporting information) | 10 $m^3$/min (fixed) |
| | 71.6 $m^3$/min (medium) | | |
| | 190.9 $m^3$/min (high) | | |
| Activity class | N/A | N/A | Spreading of liquid products |
| Emission rate | 1283.3 mg/min (Calculated by using specific gravity and a vapor pressure of cyclohexane; Spencer and Plisko, 2007) | Handling of liquids using low pressure, low speed or on medium-sized surfaces. | Spreading of liquids at surfaces or work pieces 0.3-1.0 $m^2$/hour. |
| Simulated NF concentration | 243 mg/$m^3$ (low $\beta$) | 116 mg/$m^3$ 50th percentile (370 mg/$m^3$ 75th percentile and 1960 mg/$m^3$ 95th percentile) | 2000 mg/$m^3$ 75th percentile (4600 mg/$m^3$ for 95th percentile with 95% confidence interval) |
| | 139 mg/$m^3$ (medium $\beta$) | | |
| | 127 mg/$m^3$ (high $\beta$) | | |
| Ratio of simulated and measured NF concentration* | 1.03 | 0.49 (50th percentile) | 8.51 (75th percentile) |
| | 1.01 | 0.85 (50th percentile) | 14.60 (75th percentile) |
| | 0.72 | 0.66 (50th percentile) | 11.3 (75th percentile) |

*Measured NF concentrations were for 235 mg/$m^3$ for low $\beta$, 137 mg/$m^3$ for medium $\beta$, and 177 mg/$m^3$ for high $\beta$.

**Text S4. Summary of STOFFENMANAGER® and ART studies reporting correlation coefficients**

A literature search (January 2021) was performed by using Google, Google Scholar and the PubMed search engine to identify the developmental peer-reviewed studies published after 2008 for STOFFENMANAGER® and after 2013 for ART. A systematic review by Spinazzè et al. (2019) was used to identify the relevant validation and evaluation studies, including regression and correlation analysis between estimated and observed exposure. Bayesian modeling approach was not included in this evaluation. The following keywords, including abbreviations and complete words were used:

- Stoffenmanager AND (validation OR comparison OR development) NOT nano: Resulted in 25 studies where we identified one developmental study (Schinkel et al., 2010) and eight validation and measurement comparison studies with correlation between measurements and STOFFENMANAGER® predictions (Koppisch et al., 2012; Lamb et al., 2015; E. G. Lee et al., 2019; Schinkel et al., 2010; Spinazzè et al., 2020, 2017; Tielemans et al., 2008a; van Tongeren et al., 2017).
- (Advanced REACH Tool) AND (validation OR comparison OR development): Resulted to 569 results where we identified two developmental studies (McNally et al., 2014; Sailabaht et al., 2018) that extended the multipliers for welding scenarios and five validation and measurement comparison studies with correlation between measurements and ART predictions (E. G. Lee et al., 2019; S. Lee et al., 2019; Savic et al., 2017a; Spinazzè et al., 2020, 2017)

STOFFENMANAGER® and ART calibration and validation studies show the following Spearman correlations for log-transformed data:

- STOFFENMANAGER®: Handling solids r = 0.80, volatile liquids r = 0.56, non-volatile liquids r = 0.56 (Tielemans et al., 2008a)
- STOFFENMANAGER®: Handling of powders and granules r = 0.41, Handling resulting in comminuting r = 0.69, volatile liquids r = 0.20, non-volatile liquids r = 0.63 (Schinkel et al., 2010)
- STOFFENMANAGER®: Handling r = 0.79 and machining r = 0.76 (Koppisch et al., 2012)
- STOFFENMANAGER®: Powder handling r = 0.83, volatile liquids r = 0.55, non-volatile liquids r = 0.62 (Lamb et al., 2015)
- STOFFENMANAGER®: Powder handling r = 0.68, volatile liquids r = 0.52, non-volatile liquids r = 0.47 (van Tongeren et al., 2017)
- STOFFENMANAGER®: Organic solvents r = 0.63 (Spinazzè et al., 2017)
- STOFFENMANAGER®: Handling of liquids (using low pressure, but high speed) without creating a mist or spray/haze r = 0.22, handling of liquids on large surfaces or large work pieces r = 0.22; handling of liquids on small surfaces or incidental handling of liquid r = -0.11, handling of liquids using low pressure, low speed, or on medium-sized surfaces r = 0.76, low vapour pressure (<500 Pa at room temperature) r= -0.42, medium vapor pressure (500 ≤ vapour pressure ≤ 10 000 Pa) r = -0.09, high vapor pressure (> 10 000 Pa) r = 0.60, LEV present r = 0.74 , LEV absent r = 0.23 (E. G. Lee et al., 2019)
- STOFFENMANAGER®: Inhalation Long-term exposure r = 0.287 and r = 0.228 (Spinazzè et al., 2020)
- ART: Inhalation Long-term exposure r = 0.231 and r = 0.28 (Spinazzè et al., 2020)

- ART: Organic solvents r = 0.79 (Spinazzè et al., 2017)
- ART: Vapours r = 0.61, Powders r = 0.3, Wood/stone dust r = 0.2, metal dusts r = 0.17 (Savic et al., 2017a)
- ART: Organic solvents r = 0.96 (S. Lee et al., 2019)
- ART: handling of contaminated objects or paste r = 0.59, falling liquids r = 0.89, combined two activities, handling of contaminated objects or paste and activities with relatively undisturbed surfaces (no aerosol formation) r = 0.38; spreading of liquid products r = -0.03, low vapour pressure (<500 Pa at room temperature) r = 0.88, medium vapor pressure (500 ≤ vapour pressure ≤ 10 000 Pa) r = 0.14, high vapor pressure (> 10 000 Pa) r = 0.88 (E. G. Lee et al., 2019).

Schinkel et al. (2011) did not find any correlation between ART modelled and measured values. Correlation factors were not reported by Mc Donnell et al. (2011).

## 4. References


Abadie MO, Blondeau P (2011) PANDORA database: A compilation of indoor air pollutant emissions. *HVAC&R Res*; 17: 602–613. doi: 10.1080/10789669.2011.579877

Cherrie J, Maccalman L, Fransman W *et al.* (2011) Revisiting the effect of room size and general ventilation on the relationship between near- and far-field air concentrations. *Ann Occup Hyg*; 55: 957-979. doi:10.1093/annhyg/mer092

Cherrie J, Schneider T, Spankie S *et al.* (1996) A new method for structured, subjective assessment of past concentrations. *Occup Hyg*; 3: 75–83. doi: 10.1016/S0003-4878(99)00023-X

Cherrie JW (1999) The effect of room size and general ventilation on the relationship between near and far-field concentrations. *Appl Occup Environ Hyg*; 14: 539–546. doi:10.1080/104732299302530

Cherrie JW, Fransman W, Heussen, GAH *et al.* (2020) Exposure models for REACH and occupational safety and health regulations. *Int J Environ Res Public Health*; 17: 383. doi:10.3390/ijerph17020383

Dols W, Polidoro B (2015) CONTAM User Guide and Program Documentation, Version 3.2 (No. Technical Note 1887). NIST. doi: 10.6028/NIST.TN.1887

ISES Europe (2020) Theoretical background and application of occupational exposure models. Working Group: Exposure Models. Available at: https://ises-europe.org/article/workshop-theoretical-background-occupational-exposure-models [Accessed: 11th Jan 2021]

Jayjock, MA, Armstrong T, Taylor M (2011) The Daubert Standard as Applied to Exposure Assessment Modeling Using the Two-Zone (NF/FF) Model Estimation of Indoor Air Breathing Zone Concentration as an Example. *J Occup Environ Hyg*; 8, D114–D122. doi: 10.1080/15459624.2011.624387

Koivisto AJ, Hussein T, Jayjock M (2019a) INSIGHT: Scientists claim EU uses unrealistic chemical exposure models for safety calls. Bloomberg Environment. Available at: https://news.bloomberglaw.com/environment-and-energy/insight-scientists-claim-eu-uses-unrealistic-chemical-exposure-models-for-safety-calls. [Accessed: 11th Jan 2021]

Koivisto AJ, Jensen ACØ, Koponen IK (2018) The general ventilation multipliers calculated by using a standard near-field/far-field model. *J Occup Environ Hyg*; 15: D38–D43. doi:10.1080/15459624.2018.1440084

Koontz MD, Nagda NL (1991) A Multichamber Model For Assessing Consumer Inhalation Exposure. Indoor Air 1: 593–605. doi: 10.1111/j.1600-0668.1991.00021.x

Koppisch D, Schinkel J, Gabriel S *et al.* (2012) Use of the MEGA exposure database for the validation of the Stoffenmanager model. *Ann Occup Hyg*; 56: 426–439. doi:10.1093/annhyg/mer097

Lamb J, Hesse B, Miller L *et al.* (2015) Evaluation of tier 1 exposure assessment models under REACH (eteam) Project. Research Project F2303. Dortmund/Berlin/Dresden: Bundesanstalt fur Arbeitsschutz und Arbeitsmedizin. ISBN 978-3-88261-155-7

Lee EG, Lamb J, Savic N, et al. (2019) Evaluation of Exposure Assessment Tools under REACH: Part II-Higher Tier Tools. Ann Work Expo Health 63, 230–241. doi: 10.1093/annweh/wxy098

Lee S, Lee K, Kim H (2019) Comparison of quantitative exposure models for occupational exposure to organic solvents in Korea. *Ann Work Expo Health*; 63: 197–217. doi:10.1093/annweh/wxy087

Marquart H, Heussen H, Le Deber M *et al.* (2008) ‘Stoffenmanager’, a web-based control banding tool using an exposure process model. *Ann Occup Hyg*; 52: 429-441. doi:10.1093/annhyg/men032

Mc Donnell PE, Schinkel JM, Coggins M.A *et al.* (2011) Validation of the inhalable dust algorithm of the Advanced REACH Tool using a dataset from the pharmaceutical industry. *J Environ Monit*; 13: 1597. doi:10.1039/c1em10189g

Nymark P, Bakker M, Dekkers S *et al.* (2020) Toward rigorous materials production: New approach methodologies have extensive potential to improve current safety assessment practices. *Small*; 16: 1904749. doi:10.1002/smll.201904749

Santos A, Rato R, Mafra C, Trojanova J, Sanchez V (2017) Modelling Optimization of Energy Efficiency in Buildings for Urban Sustainability: D3.5 MOEEBIUS Indoor Air Quality Assessment Models. Available at: https://ec.europa.eu/research/participants/documents/downloadPublic?documentIds=080166e5b0acb349&appId=PPGMS [Accessed: 11th Jan 2021]
Savic N, Gasic B, Schinkel J *et al.* (2017a) Comparing the Advanced REACH Tool's (ART) estimates with switzerland's occupational exposure data. *Ann Work Expo Health*; 61: 954–964. doi:10.1093/annweh/wxx069
SCCS (2018) Scientific Committee on Consumer Safety. The SCCS notes of guidance for the testing of cosmetic ingredients and their safety evaluation: 10th revision. Publications Office, LU. Available at: https://ec.europa.eu/health/sites/health/files/scientific_committees/consumer_safety/docs/sccs_o_224.pdf [Accessed: 11th Jan 2021]
Schinkel J, Fransman W, Heussen H *et al.* (2010) Cross-validation and refinement of the Stoffenmanager as a first tier exposure assessment tool for REACH. *Occup Environ Med*; 67: 125–132. doi:10.1136/oem.2008.045500
Schinkel J, Warren N, Fransman W *et al.* (2011) Advanced REACH Tool (ART): Calibration of the mechanistic model. *J Environ Monit*; 13: 1374. doi:10.1039/c1em00007a
Spinazzè A, Borghi F, Campagnolo D *et al.* (2019) How to Obtain a Reliable Estimate of Occupational Exposure? Review and Discussion of Models' Reliability. Int J Environ Res Public Health 16. doi:10.3390/ijerph16152764
Spinazzè A, Borghi F, Magni D *et al.* (2020) Comparison between communicated and calculated exposure estimates obtained through three modeling tools. *Int J Environ Res Public Health*; 17: 4175. doi:10.3390/ijerph17114175
Spinazzè A, Lunghini F, Campagnolo D *et al.* (2017) Accuracy evaluation of three modelling tools for occupational exposure assessment. *Ann Work Expo Health*; 61: 284–298. doi:10.1093/annweh/wxx004
Spencer JW, Plisko MJ (2007). A Comparison Study Using a Mathematical Model and Actual Exposure Monitoring for Estimating Solvent Exposures During the Disassembly of Metal Parts. *J Occup Environ Hyg*; 4, 253–259. doi: 10.1080/15459620701205253
Steiling W, Almeida JF, Assaf Vandecasteele H *et al.* (2018) Principles for the safety evaluation of cosmetic powders. *Toxicol Lett*; 297: 8–18. doi: 10.1016/j.toxlet.2018.08.011
Steiling W, Bascompta M, Carthew P *et al.* (2014) Principle considerations for the risk assessment of sprayed consumer products. *Toxicol Lett*; 227: 41–49. doi: 10.1016/j.toxlet.2014.03.005
Tielemans E, Warren N, Schneider T, Tischer M, Ritchie P, Goede H, Kromhout H, Van Hemmen J, Cherrie JW (2007). Tools for regulatory assessment of occupational exposure: development and challenges. *J Expo Sci Environ Epidemiol*; 17, S72–S80. doi: 10.1038/sj.jes.7500604
Tielemans E, Noy D, Schinkel J *et al.* (2008a) Stoffenmanager exposure model: Development of a quantitative algorithm. *Ann Occup Hyg*; 52:443-454. doi:10.1093/annhyg/men033
U.S. EPA, 2019. Consumer Exposure Model (CEM), User Guide Prepared for EPA Office of Pollution Prevention and Toxics by ICF under EPA Contract # EP-W-12-010. U.S. EPA Office of Research and Development, Washington, D.C.
U.S. EPA, 2018. Exposure Factors Handbook Chapter 19 (Update): Building Characteristics. (No. EPA/600/R-18/121F). U.S. EPA Office of Research and Development, Washington, D.C.
Van Tongeren M, Lamb J, Cherrie JW *et al.* (2017) Validation of lower tier exposure tools used for REACH: Comparison of tools estimates with available exposure measurements. *Ann Work Expo Health*; 61: 921–938. doi:10.1093/annweh/wxx056
